# The Bishop Index and Birth Outcomes in Nulliparous Women Undergoing Labor Induction: A Scoping Review

**DOI:** 10.3390/healthcare14152242

**Published:** 2026-07-23

**Authors:** Tito Manuel de Jesus Félix, Maria Margarida Fialho Sim-Sim, Ana Cristina Canhoto Ferrão, Maria Otília Brites Zangão

**Affiliations:** 1 Serviço de Ginecologia e Obstetrícia, Hospital CUF de Faro, 8005-226 Faro, Portugal; tito.m.felix@gmail.com; 2 Comprehensive Health Research Centre (CHRC), Escola Superior de Enfermagem São João de Deus, Universidade de Évora, 7004-516 Évora, Portugal; msimsim@uevora.pt (M.M.F.S.-S.); ana.ferrao@uevora.pt (A.C.C.F.)

**Keywords:** bishop index, labor, induced, cervical ripening, delivery, obstetric, obstetric nursing, midwifery

## Abstract

**Background:** Induction of labor is one of the most common interventions in obstetrics and continues to be associated with an increased risk of cesarean delivery among nulliparous women. The Bishop Index remains widely used at admission, but its predictive power when used in isolation is limited. Objectives: To map the available scientific evidence on the use of the Bishop Index at admission as an indicator of obstetric outcomes in nulliparous women undergoing labor induction. **Methods:** A scoping review was conducted in accordance with the Joanna Briggs Institute methodology and the PRISMA-ScR reporting guidelines. The search strategy was structured according to the PCC (Population, Concept, Context) mnemonic and covered the PUBMED (Public MEDLINE), Scopus, and Web of Science databases, as well as the CINAHL Ultimate, Medic Latina, and MEDLINE Ultimate databases on the EBSCOhost platform, with no time restrictions and limited to the English, Portuguese, Spanish, and French languages. A critical methodological appraisal was conducted as a complementary procedure to support the interpretation of the results. The protocol is registered on the OSF (Open Science Framework) platform with the DOI: 10.17605/OSF.IO/WYE24. **Results:** The 38 eligible studies were included in the overall mapping; the JBI methodological assessment was used only as a supplementary procedure to contextualize the strength of the evidence, and 21 studies with a JBI score of ≥75% were prioritized solely for an in-depth interpretive synthesis. These studies span the period from 1992 to 2026, with a diversity of study designs and geographic contexts, allowing for a characterization of the use of the Bishop Index in the process of labor induction in nulliparous women and the resulting obstetric outcomes. **Conclusions:** The evidence confirms the clinical utility of the Bishop Index as a bedside tool but reveals significant heterogeneity among studies, definitions of success, and induction methods. When reported, predictive performance indicators tended to favor combined models and ultrasound parameters over the use of the Bishop Index alone. The most recent studies point to the potential of ultrasound parameters and combined models to improve the predictive capacity of obstetric outcomes.

## 1. Introduction

In general, labor induction involves the artificial stimulation of uterine contractions before the spontaneous onset of labor, with the goal of achieving vaginal delivery when the benefits of terminating the pregnancy outweigh the risks of continuing it. Labor induction is, therefore, an intervention aimed at triggering a process that has not occurred spontaneously by the end of the pregnancy. A full-term pregnancy is defined as one ranging from 39 weeks and 0 days of gestation to 40 weeks and 6 days of gestation, in accordance with the classifications endorsed by the American College of Obstetricians and Gynecologists and the Society for Maternal-Fetal Medicine, which define pre-term (37 weeks and 0 days of gestation through 38 weeks and 6 days of gestation), full-term (39 weeks and 0 days of gestation through 40 weeks and 6 days of gestation), late-term (41 weeks and 0 days of gestation through 41 weeks and 6 days of gestation), and post-term (42 weeks and 0 days of gestation or more) to more accurately describe births occurring at 37 weeks and 0 days of gestation or more [1,2]. However, gestational age is not the only criterion for labor induction: conditions such as Fetal Growth Restriction, Diabetes, Gestational Hypertension, or prolonged pregnancy may justify early delivery, provided that a careful assessment of the maternal-fetal risk-benefit balance is conducted. In this context, labor induction is the safest option to prevent any degree of maternal and fetal morbidity and mortality.

In this regard, the decision to induce labor is based on a careful assessment of various factors, and its success depends largely on the characteristics of the cervix and the method used, with success defined as a vaginal delivery as the outcome of induction; in turn, failed induction is associated with a higher probability of cesarean delivery, longer hospital stays, and the use of more invasive interventions with high maternal-fetal risk [3,4]. According to the recommendations issued by the World Health Organization [5], the decision to induce labor should be based on a careful assessment of the risk-benefit balance, as well as the maternal, cervical, and fetal conditions.

Induction of labor is currently one of the most common obstetric interventions worldwide, with rates ranging from 20% to 30% of births in developed countries [6], with a progressive increase observed over the past decades, specifically from 9.5% in the 1990s to 22.1% in 2004 [7]. Labor induction continues to increase in many health care systems and remains central to the contemporary organization of obstetric care. Recent data highlight that the cesarean section rate among nulliparous women with a singleton, term, cephalic-presentation pregnancy remains an important indicator of quality of care, standing at approximately 25.6% in the United States, while current international recommendations emphasize the systematic documentation of cervical assessment and the selection of the induction method based on cervical ripening [8,9,10].

The Bishop Index was developed in the 1960s, when obstetrician Edward H. Bishop proposed a clinical “pelvic scoring” system to assess more objectively and consistently whether the cervix is favorable and, consequently, the likelihood of success for elective labor induction. Published in 1964 in the journal Obstetrics & Gynecology [11] the original model systematized the evaluation of the cervix using five parameters obtained through vaginal examination, on a scale of 0–13 points (dilatation, effacement, consistency, cervical position, and plane of presentation), with the aim of supporting clinical decision-making and reducing inductions with a low probability of progressing to vaginal delivery. A simplified version of the Bishop index [12] was later proposed, ranging from 0 to 9 points and based solely on cervical dilation, effacement, and fetal presentation, downplaying the importance of cervical position and consistency. The results showed that the simplified Bishop Index had predictive power similar to that of the original score in estimating vaginal delivery after induction, which justifies its use to this day.

At admission for labor induction, cervical assessment using the Bishop Index [13] is the primary clinical criterion for determining the induction method to be used: cervical ripening for unfavorable cervixes with a Bishop Index of 6 or less, or oxytocic perfusion for favorable cervixes with a Bishop Index of 6 or more, with special emphasis on nulliparous women, in accordance with institutional protocol [14]. This score is, in itself, an important clinical tool for successful induction available to healthcare professionals [15]. However, the Bishop Index should not be interpreted in isolation, since maternal and obstetric factors, such as parity, gestational age, body mass index, maternal age, and estimated fetal weight, can influence the success of induction and the mode of delivery [13,16,17].

Thus, the objective of this systematic review is to map the available scientific evidence regarding the use of the Bishop Index at admission as a predictor of obstetric outcomes in nulliparous women undergoing labor induction.

## 2. Materials and Methods

A scoping review seeks to systematically and comprehensively map the available evidence on a topic, identifying key concepts, types of evidence, knowledge gaps, and areas for future research [18,19]. This review was conducted according to the Joanna Briggs Institute (JBI) methodology and the PRISMA-ScR 2020 reporting guidelines [20].

Assessing the methodological quality of primary studies using standardized tools is not a mandatory requirement in a scoping review [21]. Critical assessment of methodological quality was not used as an exclusion criterion for eligible studies; rather, it was adopted as a complementary procedure to guide the interpretation of results and to distinguish between an in-depth synthesis and an overview of the evidence. Authors may decide to assess the reliability and rigor of the methodology of the included studies, taking into account the objective of the review [22,23].

The protocol is registered on the OSF (Open Science Framework) platform with the DOI: https://doi.org/10.17605/OSF.IO/WYE24.

### 2.1. Identification of the Research Question

In this context, this scoping review is guided by a research question defined according to the PCC (Population–Concept–Context) model, with the aim of mapping the available scientific evidence on the topic under study (Table 1).

Research question: What evidence is available regarding the use of the Bishop Index at admission as a predictor of obstetric outcome in nulliparous women undergoing labor induction?

### 2.2. Identification of Relevant Studies

The identification of relevant studies was based on a search strategy aligned with the research question and structured according to the PCC model. The main strategy prioritized broad combinations of terms related to the Bishop Index, labor induction, cervical ripening, and obstetric outcomes; the terms “obstetric nursing” and “midwifery” were used only as sensitivity searches and not as mandatory filters. The search was conducted in the PUBMED, Scopus, and Web of Science databases, as well as in the CINAHL Ultimate, Medic Latina, and MEDLINE Ultimate databases via EBSCOhost. The restriction to the English, Portuguese, Spanish, and French languages was maintained for operational reasons and is acknowledged as a methodological limitation. The complete strategy for each database is presented in Table 2.

### 2.3. Selection of Studies

The third stage of the scoping review involved the study selection process, in which the identified records were evaluated based on predefined inclusion and exclusion criteria aligned with the PCC formulation of the research question, as presented in Table 3.

To clarify the inclusion criteria, in line with current guidelines for the management of low-risk pregnancies and normal childbirth, we defined “low risk” as a singleton pregnancy, cephalic presentation, and the absence of any immediate contraindication for vaginal delivery. Nevertheless, to remain true to the review’s mapping objective, we did not automatically exclude studies involving induction for post-term pregnancy or for common obstetric indications when these remained relevant to cervical assessment at admission and to the outcomes of induction.

The exclusive focus on nulliparous women remains clinically justified, as this group is more likely to require pro-longed labor induction, has a greater need for cervical ripening, and faces an increased risk of cesarean section. Observational cohort studies have consistently identified nulliparity as an independent predictor of cesarean section, even after adjusting for maternal, fetal, and obstetric factors [24,25].

Unlike multiparous women, whose previous obstetric history strongly influences the outcome of delivery, nulliparous women have no prior deliveries that might influence cervical response to induction or the progression of labor. The absence of prior obstetric experience eliminates an important confounding factor, allowing for a more isolated and accurate assessment of the predictive value of the Bishop Index. Studying nulliparous women in the context of labor induction offers significant methodological and clinical advantages, as this group constitutes a more homogeneous population with a higher risk of induction failure and cesarean delivery [26].

**Figure 1:**
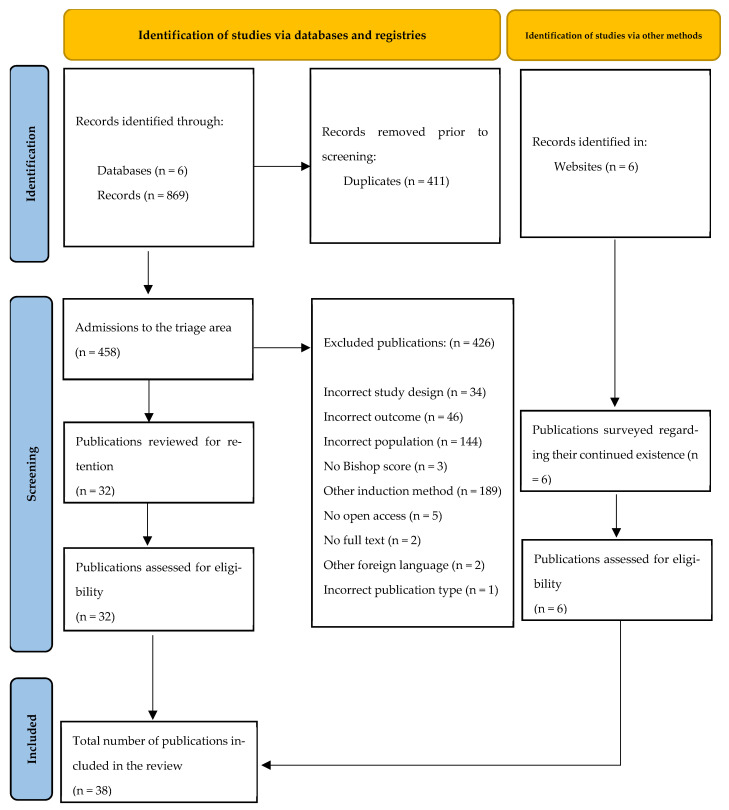
JBI Prisma flowchart of the selection process (Adapted from [20,28]).

After the search, the records were exported to the RAYYAN bibliographic management system [27], where duplicates were removed. Screening by title and abstract was performed independently by two reviewers, followed by a full reading of the potentially eligible articles. Disagreements were resolved by consensus without the need for a third reviewer. Whenever possible, data specific to nulliparous women were given priority. The process of identification, screening, eligibility, and inclusion was organized in accordance with PRISMA-Scr 2020 (Figure 1).

### 2.4. Methodological Quality Assessment–JBI

After identifying the 38 eligible articles, a critical methodological assessment was conducted using the JBI tools, not as a defining criterion for eligibility, but as a complementary strategy to contextualize the methodological robustness of the included studies. All 38 studies remained in the overall evidence mapping. Studies with a JBI score of ≥75% were merely prioritized for an in-depth interpretive synthesis; this should be understood as a stratification of methodological robustness within a scoping review and not as the selective exclusion typical of a systematic review. The methodological quality appraisal of the 38 eligible studies has been added to Table S1.

## 3. Results

This of the 38 eligible articles, 21 were prioritized for in-depth synthesis because they had a JBI score of 75% or higher. This decision did not alter the overall overview of the evidence: the 38 studies continued to be included in the characterization of the field, while Table 4 focused on the narrative synthesis of the most methodologically robust studies. No meta-analysis or pooled calculations of Area Under the Curve (AUC) or Odds Ratio (OR) were performed due to the heterogeneity of the study designs, definitions of success, and reported outcomes. Data extraction was organized to collect information relevant to answering the research question, as presented in Table 4, including, where applicable, the level of evidence according to the JBI classification.

### 3.1. Sample Characteristics

The prioritized subset includes 21 studies published between 1992 and 2026, spanning a period of 34 years. The temporal distribution reflects the inclusion of both classic and contemporary research on cervical ripening, labor induction, and the predictive power of the Bishop Index. There is a greater concentration of studies in the most recent period, with 8 studies published between 2020 and 2026, 6 studies between 2010 and 2019, and 7 studies between 1992 and 2009. This diversity enhances the descriptive value of the review, but it also introduces significant clinical heterogeneity, particularly with regard to the induction method, gestational age, definition of success, admission criteria, and sample composition.

In terms of geographic origin, the studies are distributed internationally, including the United States, China, Japan, Germany, France, Israel, Spain, Greece, Italy, the Netherlands, and Turkey. The countries with the highest representation are the United States, with 4 studies, followed by China and Japan, with 3 studies each. Germany, France, and Israel each have 2 studies, while Spain, Greece, Italy, the Netherlands, and Turkey each contribute 1 study.

Methodologically, observational and cohort studies, both prospective and retrospective, predominate alongside randomized controlled trials. According to the JBI classification, 6 studies have evidence level 1.c, 11 studies have level 3.e, and 4 studies have level 4.b. This distribution indicates an evidence base composed primarily of observational studies but with a significant presence of experimental or quasi-experimental studies of greater methodological robustness (Table 5).

Methodological quality, assessed using JBI tools, was rated as high in 19 studies and moderate in 2 studies within the in-depth synthesis. Nevertheless, the direct comparability of the results remains limited by methodological and clinical heterogeneity; therefore, the conclusions should be interpreted in a narrative and contextual manner.

In summary, the 21 prioritized studies made it possible to identify consistent trends regarding the use of the Bishop Index at admission for labor induction in nulliparous women, but they do not allow for aggregated quantitative inferences. The final interpretation depends both on methodological robustness and on variability across populations, protocols, and definitions of success.

### 3.2. Key Findings

The evidence reviewed demonstrates a continued interest in predicting the success of labor induction, rather than merely comparing methods. In several studies, the primary outcome was the probability of vaginal delivery, the time from induction to delivery, vaginal delivery within 24 h, or the need for a cesarean section, showing that the Bishop Index was used primarily as a clinical predictive marker.

The Bishop Index continues to emerge as a benchmark clinical tool for cervical assessment at admission. However, when performance metrics were reported, the results showed only moderate predictive ability, often inferior to that of complementary approaches: for example, Michail et al. [3] reported an AUC of 0.784 for the Bishop Index versus 0.891 for a composite ultrasound score [43], and Uyar et al. found that ultrasound-measured cervical length (AUC 0.819) performed better than the Bishop Index [44].

Although some studies include mixed populations, nulliparity consistently emerges as a clinical context associated with greater vulnerability to prolonged labor and cesarean section. Whenever the data were not fully stratified by nulliparity, this aspect was explicitly considered in the interpretation of the results and discussed as a limitation [26,30,32,46].

The pharmacological options presented in the analyzed studies are varied, with the predominant use of prostaglandins and oxytocin described in different dosages and formulations. Prostaglandin E2 (PGE2) is the most frequently mentioned pharmacological agent and is administered in the form of a controlled-release pessary (10 mg); it is considered safe, with easily reversible hyperstimulation after removal, and is particularly successful in nulliparous women [36,38,48], followed by intracervical gel, both for cervical ripening in unfavorable cervixes (with a low Bishop Index). Prostaglandin E1 is also mentioned, and its efficacy is considered high, despite the associated risks of uterine hyperstimulation and difficulty in controlling its absorption and reversing its effects [39,42]. Oxytocin is the pharmacological option reserved for high Bishop Index scores, greater than 6, without the need for cervical ripening, and there are several protocols for its administration. Induction methods were included in the synthesis as the clinical context necessary for interpreting the predictive performance of the Bishop Index. Thus, the comparison between dinoprostone, oxytocin, misoprostol, or mechanical methods is not presented as the primary objective of the review, but rather as an explanatory variable for the heterogeneity of the observed outcomes.

Some authors cite the mechanical Cook Balloon method as a safe alternative for avoiding the risks of uterine hyperstimulation, noting the OBLIGE Study, which also offers the added advantage of allowing cervical ripening to be performed on an outpatient basis [45].

## 4. Discussion

Labor induction is a fundamental intervention in contemporary obstetric practice. Labor induction is a fundamental intervention in contemporary obstetric practice. In addition to the sustained increase in its use, current care indicators show that the cesarean section rate among nulliparous women with a singleton, term, cephalic presentation pregnancy remains high, reinforcing the need for reliable clinical triage tools at admission [8]. At the same time, recent NICE guidelines and other clinical recommendations continue to regard documented cervical assessment and the choice of induction method based on cervical ripening as central elements of decision-making [9,10].

The predictive ability regarding obstetric outcomes after induction depends, in part, on the tool used at admission. Although the Bishop Index remains widely used, its semiquantitative nature and reliance on vaginal examination limit interobserver reproducibility and prognostic accuracy, especially in cases of very unfavorable cervixes. Furthermore, the included studies differ substantially in terms of induction methods, gestational age, definitions of success, clinical protocols, and care settings, which provides a plausible explanation for the seemingly inconsistent results.

The superiority of highly objective and mathematical methods is advocated as a sound alternative: The Novel Ultrasound-based Induction Score demonstrates significantly greater predictive power than the traditional Bishop Index. It is based on ultrasound assessment of biometric parameters such as cervical length, fetal presentation, head-to-perineum distance, elastography, and cervical angulation [41]. According to the author, this new scoring method is a composite scoring system designed to objectively quantify cervical ripening and fetal presentation for labor induction. Each parameter is scored from 0 to 2, resulting in a total of 10; the higher the score, the greater the probability of successful induction, stratified as follows: low favorability (score 0–3), intermediate (score 4–6), and high (score 7–10). It applies to both nulliparous and primiparous women.

The LaborPro system, which integrates measurement of cervical length and fetal presentation using ultrasound, significantly outperformed the Bishop Index in predicting the success of induction, particularly in nulliparous women [47]. Specifically, ultrasound assessment of the level of presentation proved to be the most consistent predictor of delivery within 24 h in nulliparous women. This system is described as a tool based on objective ultrasound findings regarding cervical length, fetal station, and level of presentation. The system allows for the determination of spatial coordinates in real time with an accuracy of 1.0 mm through the strategic placement of various sensors. Wu’s study demonstrated that a model integrating objective ultrasound data with clinical assessment of cervical consistency exhibits a predictive ability for the success of induction (vaginal delivery within 24 h) that is significantly superior to that of the traditional Bishop Index. The author also notes that ultrasound assessment of the level of presentation is a robust independent predictor: each centimeter observed in the descent of the presentation increases the probability of vaginal delivery fivefold. It is further emphasized that this rigorous and objective system, applied to the management of labor induction in nulliparous women, helps reduce the incidence of unnecessary cesarean sections.

Another study compared transvaginal ultrasound measurements of cervical length in the supine and upright positions to predict the need for cesarean section in nulliparous women. Changing from the supine to the upright position can provide additional information about the relationship between fetal presentation and the pelvic inlet, with cervical length measured in the upright position showing better predictive ability for induction failure than the traditional Bishop Index [40]. The author suggests that cervical length measurement should first be performed with the woman in the supine position (for at least 5 min) and then repeated in the upright position (at least 1 min after the change in position). The change in position serves as a functional test. The upright position allows for better assessment of fetal head engagement and can eliminate cervical funneling, which is sometimes masked when the woman is lying down. Among the ultrasound variables, only cervical length measured in the upright position was a significant predictor of the need for cesarean section in nulliparous women. Each 1 mm increase in this length was associated with a 14% increase in the probability of cesarean section. Cervical length measured by ultrasound (in both positions) was shown to have greater predictive power for induction failure (need for cesarean section due to lack of progress) than the traditional Bishop Index. These findings reinforce the potential utility of ultrasound as a complement to clinical assessment.

A critical review of the body of studies reveals a gradual shift from exclusively clinical and subjective models toward integrated and more objective approaches. The Bishop Index remains clinically relevant due to its simplicity, speed, and availability at the bedside, but the reviewed literature consistently suggests that its performance improves when interpreted in conjunction with other maternal, obstetric, and ultrasound variables.

Given the limitations associated with the traditional Bishop Index, several authors have described alternative or modified classification systems to predict the success of labor induction, particularly in cases of highly unfavorable cervical conditions. Among these is the Hughey Score, originally proposed by Hughey et al. in 1976 [32], which adds parity to the cervical assessment. The Levine Score, developed in 2018, is a model that integrates the modified Bishop Index (cervical dilation, effacement, and level of presentation) with anthropometric and obstetric variables: height, BMI, parity, and gestational age [32]. It is considered one of the most accurate classification processes for predicting the success of labor induction in nulliparous women with unfavorable cervixes, with success defined as a vaginal delivery. In general, these models suggest that the prediction of vaginal delivery in nulliparous women improves when cervical status is interpreted in conjunction with maternal and fetal characteristics.

The recognition that labor induction has failed and its impact on the cesarean section rate, particularly among nulliparous women, is a much-discussed topic today. Nulliparity and an unfavorable cervical condition are consistently associated with a lower probability of vaginal delivery following induction [26,34]. However, the interpretation of risk should not overlook additional maternal and obstetric factors. The evidence is unanimous in considering nulliparity alone as a significant risk factor for failed induction, associated with longer latent phases and a greater need for prior cervical ripening, suggesting that variables such as maternal age, BMI, gestational age, estimated fetal weight, and method of induction may modify the observed effect of the Bishop Index on the obstetric outcome.

Therefore, the choice of the most appropriate induction method for nulliparous women should strike a balance between efficacy, maternal safety, and neonatal safety. The Cook Balloon is cited as a highly effective alternative mechanical method in nulliparous women, reducing the time interval between induction and delivery by 7 h when compared to prostaglandin E2, without increasing the risk of cesarean section, that is, without compromising the success of induction [31,45,46], the Oblige 2025 study [45] introduces a fundamental element to the discussion regarding the process, due to the lower propensity for uterine hyperstimulation and the possibility of using the mechanical method on an outpatient basis without the need for hospitalization. Although this method does not reduce overall cesarean section rates, the opportunity it provides for the pregnant woman to remain safely in the comfort of her home during the cervical ripening phase is not only innovative but also appealing from the perspective of humanizing the care provided. In turn, pharmacological methods using dinoprostone remain clinically relevant, especially in cases of unfavorable cervical conditions, although they require adequate monitoring and a contextualized comparison with mechanical methods [36,37,38].

The most recent studies reinforce the interest in ultrasound models and integrated systems for cervical and fetal assessment. This trend is consistent with recent obstetric evidence outside the specific field of induction, Yalcin et al. demonstrated that early inflammatory biomarkers lose their independent predictive value after adjustment for clinical and metabolic factors, supporting the idea that isolated markers should be integrated into multiparametric models rather than used as standalone predictors [49].

It is important to acknowledge additional methodological limitations of this review. Prioritizing studies with an evidence-based practice index (JBI) of ≥75% improves interpretive consistency but partially limits the scope of the review beyond that of a classic systematic review. Furthermore, the restriction to four languages and the use of a sensitivity search with additional terms related to obstetric nursing and midwives may have contributed to the omission of some relevant literature. Therefore, the results should be interpreted as a critical and narrative mapping of the evidence, rather than as a definitive quantitative synthesis.

## 5. Conclusions

The reviewed evidence supports the view that the Bishop Index continues to have clinical utility in the admission of nulliparous women for labor induction, primarily because it is simple, accessible, and quick to apply. However, this utility should not be confused with universal predictive superiority: its performance is variable, and when used in isolation, it is often matched or surpassed by ultrasound parameters and combined models.

Thus, the Bishop Index should be understood as a component of a broader clinical evaluation rather than as a definitive predictor of the success of induction. The most recent evidence supports the integration of vaginal examination, maternal characteristics, gestational age, body mass index, and ultrasound parameters—particularly cervical length and fetal presentation.

Failure of induction and the occurrence of cesarean delivery result from a multifactorial interaction. Nulliparity remains an important risk factor, but its interpretation must be contextualized by maternal age, body mass index, indication for induction, gestational age, estimated fetal weight, and the protocol used.

For clinical practice, the results of this review reinforce the need to standardize cervical assessment, recognize interobserver variability in vaginal examination, and incorporate multivariate models whenever available. The choice of induction method should remain individualized and informed by the clinical context, rather than depending exclusively on a single score.

Another important point highlights the need for healthcare professionals to consider Body Mass Index and nulliparity as factors that require closer monitoring and a different approach to managing expectations, given the increased risk of induction failure.

This awareness on the part of healthcare professionals allows for a clearer understanding that induction failure is influenced not only by the therapeutic protocol but, above all, by the intrinsic characteristics of the pregnant woman.

It is also worth highlighting the need to value the latency period as a crucial strategy, not to be interpreted as induction failure, provided there is no maternal-fetal risk.

Finally, the key role of the healthcare professional in monitoring before and during the induction process is highlighted, involving direct interventions to control and reverse associated adverse effects.

Future research should clarify in which clinical subgroups the combination of clinical cervical assessment and ultrasound parameters offers the greatest predictive benefit, as well as report metrics such as AUC, adjusted ORs, and standardized definitions of successful induction more consistently.

## Figures and Tables

**Table 1:** PCC Model.

Component	Element	Description
P	Population	Low-risk nulliparous pregnant women undergoing labor induction.
C	Concept	Bishop Index and labor induction.
C	Context	Obstetrics Services/Delivery Room (Admission for induction).

**Table 2:** Results of the search conducted on 22 January 2026, at 6:30 P.M.

Database	Search Criteria	Filters Applied	Number of Studies Found
EBSCO (MEDLINE Ultimate + CINAHL Ultimate + Medic Latina)	S1: Bishop score AND labor, induced AND cervical ripening AND delivery obstetric AND (obstetric nursing OR midwifery)	Full Text	0
	S2: Bishop score AND labor, induced AND cervical ripening AND delivery obstetric AND obstetric nursing	Full Text	130
PUBMed	S1: Bishop score AND labor, induced AND cervical ripening AND delivery obstetric AND (obstetric nursing OR midwifery)	Full Text	0
	S2: Bishop score AND labor, induced AND cervical ripening AND delivery obstetric AND obstetric nursing	Full Text	0
	S3: Bishop score AND labor, induced AND cervical ripening AND delivery obstetric	Full Text	523
Scopus	S1: Bishop score AND labor, induced AND cervical ripening AND delivery obstetric AND (obstetric nursing OR midwifery)	Full Text	0
	S2: Bishop score AND labor, induced AND cervical ripening AND delivery obstetric AND obstetric nursing	Full Text	0
	S3: Bishop score AND labor, induced AND cervical ripening AND delivery obstetric	Full Text	193
Web of Science	S1: Bishop score AND labor, induced AND cervical ripening AND delivery obstetric AND (obstetric nursing OR midwifery)	Full Text	0
	S2: Bishop score AND labor, induced AND cervical ripening AND delivery obstetric AND obstetric nursing	Full Text	0
	S3: Bishop score AND labor, induced AND cervical ripening AND delivery obstetric	Full Text	23

**Table 3:** Inclusion and Exclusion Criteria.

Criterion	Description
Population	Studies discussing aspects related to low-risk nulliparous pregnant women undergoing labor induction.
Concept	Studies that mention the Bishop Index as a clinical tool in the labor induction process.
Context	Studies that refer to labor induction as a process in a hospital setting.
Inclusion Criteria	Studies in English, Portuguese, Spanish, and French. For the in-depth synthesis, articles with a JBI methodological rating of 75% or higher were prioritized.
Exclusion Criteria	Opinion pieces, secondary studies, letters to the editor, and studies that do not establish a relationship between the Bishop Index score and the labor induction process. Gray literature and other formulations of PGE2 were not included in this review.

**Table 4:** Extraction of narrative data from the 21 studies selected for in-depth interpretation.

Author Year	Article Title JBI Level of Evidence	Type of Study	Objetive	Population	Initial Assessment	Induction Method	Obstetric Outcome/Duration of Labor	Type of Delivery	Key Findings
Ashwal et al., 2017 [29]	Effect of fetal gender on induction of labor failure rates Level 3.e	Retrospective cohort analysis	To assess the effect of fetal sex on labor induction failure rates, stratified by indication.	1062 singleton pregnancies (34–42 weeks) that underwent cervical ripening.	Bishop Index ≤ 7.	Vaginal device for the controlled release of PGE2 (Propess 10 mg).	Failure rate of 20.1%; no significant differences in the interval between induction and delivery by gender.	14.3% cesarean section; no significant differences between male and female fetuses.	The failure rate of induction is not affected by fetal sex, regardless of the indication for induction.
Chen et al., 2014 [30]	Evaluation of Propess outcomes for cervical ripening and induction of labour in full-term pregnancy Level 1.c	Comparative randomized clinical study	To compare the efficacy and safety of vaginal Propess with intravenous oxytocin for cervical ripening and labor induction.	169 primiparous women with term pregnancy (37–41 weeks) and Bishop Index ≤ 6.	Vaginal examination to calculate the Bishop Index (consistency, length, dilation, position, and presentation level).	Propess (dinoprostone 10 mg) vs. Oxytocin (low dose: 2.5 IU in 500 mL).	Induction-to-delivery interval: 15.8 h (Propess I) and 10.5 h (Propess R) vs. 31.4 h (Oxytocin); total labor time shorter in the Propess group.	81.8% vaginal delivery in the Propess group vs. 35.6% in the oxytocin group.	Propess is an effective and safe approach to promoting cervical ripening and inducing labor, with higher success rates than oxytocin.
Sherman et al., 2001 [31]	Balloon Cervical Ripening With Extra-Amniotic Infusion of Saline or Prostaglandin E2: A Double-Blind, Randomized Controlled Study Level 1.c	Double-blind randomized controlled study	To compare extra-amniotic infusion of diluted PGE2 solution with saline infusion in balloon cervical ripening.	116 women with pregnancy complications and Bishop Index ≤ 3.	Cardiotocographic monitoring and pre-ripening Bishop Index.	Balloon (30 mL Foley catheter) with PGE2 infusion (0.5 mg/mL) vs. balloon with saline.	Balloon expulsion time: 4.7 h (PGE2) vs. 6.5 h (saline); ripening-to-delivery interval: 17.5 h (PGE2) vs. 22.0 h (saline).	Cesarean rates: 10% (PGE2) vs. 17% (saline); no statistically significant difference.	Extra-amniotic balloon ripening with PGE2 infusion is faster and more effective than saline, resulting in more spontaneous deliveries.
Tollon et al., 2022 [32]	Prediction of successful labor induction with very unfavorable cervix: A comparison of six scores Level 3.e	Secondary analysis of a prospective multicenter observational cohort study	To compare the ability of six scoring systems to predict successful labor induction in women with Bishop Index < 3.	600 women with Bishop Index < 3 prior to cervical ripening.	Assessment of all Bishop Index parameters and maternal characteristics (BMI, height, parity).	Cervical ripening with prostaglandins, cervical balloon, or vaginal misoprostol.	Duration between ripening and delivery: median 37.21 h for vaginal delivery and 33.21 h for cesarean section.	68% (408/600) vaginal delivery.	Scores that include parity (simplified Bishop with parity, Hughey or Levine) are more accurate at predicting success with highly unfavorable cervixes.
Zhao et al., 2019 [17]	Prediction of the induction to delivery time interval in vaginal dinoprostone-induced labor: a retrospective study in a Chinese tertiary maternity hospital Level 3.e	Retrospective clinical research study	To investigate the potential factors affecting the time interval between induction and delivery in women induced with vaginal dinoprostone.	1265 women undergoing labor induction with dinoprostone (PGE2)	Bishop Index < 6.	Vaginal dinoprostone device (Propess 10 mg).	Mean induction-to-delivery interval: 18.92 ± 12.50 h.	76.3% vaginal delivery.	Gestational age, parity, and fetal weight are the main predictive factors of the induction-to-delivery time interval.
Droulez et al., 2008 [33]	Prédiction de la réussite du déclenchement du travail. Comparaison entre le score de Bishop et le dosage de la fibronectine fœtale Level 3.e	Non-randomized descriptive prospective cohort study	To compare the Bishop score and fetal fibronectin in predicting success of labor induction within 24 h.	234 pregnant women with singleton pregnancy, gestational age ≥ 37 weeks, intact membranes, and cephalic presentation.	Vaginal fetal fibronectin assay followed by Bishop score determination.	Prostaglandin E2 (2 mg) or Oxytocin.	Success (vaginal delivery ≤ 24 h) in 77.3%. Mean induction time: 7.25 h (FFN+) vs. 9.11 h (FFN−).	Vaginal (66.2%), Instrumental (11.1%), Cesarean (22.6%).	Fetal fibronectin alone is not superior to the Bishop Index in predicting induction success.
Gerli et al., 2013 [34]	Single indications of induction of labor with prostaglandins and risk of cesarean delivery: A retrospective cohort study Level 4.b	Retrospective cohort study	To determine the risk of cesarean section following prostaglandin induction according to the indication for induction.	324 pregnant women undergoing pharmacological labor induction with dinoprostone.	Bishop score, maternal age, BMI, parity, and gestational age.	Dinoprostone (0.5 mg intracervical gel or 10 mg controlled-release vaginal device).	Mean labor duration: 217 min (overall median).	Vaginal (60.4%), Instrumental (0.01%), Cesarean (40.5%).	Only post-term pregnancy significantly increased the risk of cesarean section; advanced maternal age and low Bishop Index are risk factors.
Gonen et al., 1998 [35]	Prediction of successful induction of labor: comparison of transvaginal ultrasonography and the Bishop score Level 3.e	Consecutive prospective cohort study	To determine whether cervical transvaginal ultrasound is a better predictor of success than the Bishop score.	86 pregnant women scheduled for labor induction.	Digital examination (Bishop Index) and transvaginal ultrasound (cervical length).	Oxytocin or intracervical prostaglandin E2 gel.	Successful induction (vaginal delivery on the day of admission) in 54/86 cases. Mean labor duration: 34 h (success) vs. 28 h (failure).	Vaginal (62.8%), Cesarean (13.9% of total; 11 due to failure to progress).	Transvaginal ultrasound did not improve the prediction obtained by the Bishop Index; the Bishop Index and parity were the only independent predictors.
Imai et al., 2023 [36]	Comparison of the efficacy between controlled-release dinoprostone delivery system (PROPESS) and Cook’s double balloon catheter plus oxytocin: A retrospective single-center study in Japan Level 4.b	Single-center retrospective study	To compare the efficacy of the PROPESS system vs. Cook’s double balloon catheter (DBC) plus oxytocin.	197 term pregnant women with unfavorable cervix (PROPESS = 113, DBC = 84).	Bishop score ≤ 6, singleton term cephalic pregnancy.	PROPESS system (10 mg) vs. Cook Double Balloon Catheter (80 mL) + Oxytocin.	Ripening success at 24 h: 53.1% (PROPESS) vs. 36.9% (DBC). Mean treatment time: 7.0 h.	Vaginal delivery: 75.2% (PROPESS) vs. 59.5% (DBC).	PROPESS showed advantages in vaginal delivery rate and 24 h cervical ripening compared to DBC + oxytocin.
Imai et al., 2023 [37]	Clinical significances of Bishop score and vaginal bleeding to controlled-release dinoprostone delivery system (PROPESS) efficacy for cervical ripening: A retrospective single-center study in Japan Level 4.b	Retrospective case–control study	To assess the effect of vaginal bleeding on PROPESS efficacy and associated factors.	100 term pregnant women undergoing labor induction with PROPESS for unfavorable cervix.	Bishop score ≤ 6, vaginal pH (in 24 selected cases).	Controlled-release dinoprostone delivery system (PROPESS) for up to 12 h.	Ripening success (Bishop ≥ 7) in 25%. Treatment time: 6.0 h (effective group) vs. 7.0 h (ineffective group).	Vaginal delivery: 92% (effective group) vs. 69.3% (ineffective group).	The Bishop Index and the presence of vaginal bleeding at insertion (which raises vaginal pH) are predictors of success.
Itoh et al., 2021 [38]	Efficacy and safety of controlled-release dinoprostone vaginal delivery system (PROPESS) in Japanese pregnant women requiring cervical ripening: Results from a multicenter, randomized, double-blind, placebo-controlled phase III study Level 1.c	Multicenter, randomized, double-blind, placebo-controlled study	To evaluate the efficacy and safety of the controlled-release dinoprostone vaginal pessary in Japanese women.	114 Japanese pregnant women (57 PROPESS, 57 placebo) at 41 weeks with Bishop Index ≤ 4.	Bishop Index ≤ 4, fetal monitoring (CTG) and vital signs.	PROPESS device (10 mg controlled-release dinoprostone) vs. Placebo for 12 h	Ripening success at 12 h: 47.4% (PROPESS) vs. 14.3% (Placebo). Mean time to vaginal delivery: 26.18 h (PROPESS) vs. 33.02 h (Placebo).	Vaginal (81.6% PROPESS vs. 84.6% Placebo), Cesarean (18.7% vs. 13.9%).	Administration of PROPESS for 12h is effective for cervical ripening and reduces the time to vaginal delivery.
López Jiménez et al., 2023 [16]	Prediction of an effective cervical ripenning in the induction of labour using vaginal dinoprostone Level 3.e	Observational prospective study	To develop a predictive model for the success of cervical ripening with PROPESS.	204 pregnant women requiring labor induction in Spain.	Bishop Index ≤ 6 and cervical length by ultrasound.	Vaginal dinoprostone (PROPESS 10 mg) slow-release for up to 24 h.	Effective cervical ripening (Bishop > 6) in 44.6%. Time with PROPESS: 9.3 h (success) vs. 14.5 h (failure).	Spontaneous (55.4%), Instrumental (16.6%), Cesarean	Model C is useful for predicting the success of cervical ripening
Mayer et al., 2016 [39]	Initial clinical experience with a misoprostol vaginal insert in comparison with a dinoprostone insert for inducing labor Level 4.b	Retrospective cohort study	To compare vaginal misoprostol tablet (200 µg) with vaginal dinoprostone pessary (10 mg controlled release).	243 pregnant women (Misoprostol = 119, Dinoprostone = 124) ≥ 36 weeks.	Modified Bishop score, BMI, parity, and fetal membranes.	Vaginal Misoprostol (200 µg) vs. Dinoprostone (10 mg).	Vaginal delivery at 24 h: 77.3% (M) vs. 74.2% (D). Time to vaginal delivery: 761.76 min (M) vs. 805.17 min (D).	Vaginal (approx. 89–90%), Instrumental (10.1% vs. 7.3%), Cesarean (10.1% vs. 10.5%).	Both are equivalent in terms of 24h vaginal delivery rate and neonatal safety.
Meijer-Hoogeveen et al., 2009 [40]	Transvaginal ultrasound measurement of cervical length in the supine and upright positions versus Bishop score in predicting successful induction of labor at term Level 3.e	Observational prospective study	To assess the predictive value of cervical length in the supine and upright positions vs. Bishop score.	102 pregnant women (68 nulliparous, 34 multiparous) at term.	Transvaginal ultrasound (supine and upright) and digital examination (modified Bishop score).	Oxytocin or intravaginal prostaglandin E2 (if Bishop < 6).	Median induction-to-delivery interval: 6 h (range 1–127 h). Success (vaginal delivery) in 85%	Vaginal (85%), Cesarean (15%)	Cervical length measured in the upright position was the best predictor of cesarean section in nulliparous women.
Michail et al., 2026 [41]	Evaluation of the Bishop Score in Comparison With Ultrasonographic Markers in Predicting Successful Induction of Labor Level 3.e	Prospective observational cohort study	To compare the Bishop Index with a new composite index based on vaginal ultrasound.	200 pregnant women with gestational age ≥ 34 weeks undergoing labor induction.	Bishop Index (dilation, effacement, consistency, position, and presentation level) and composite ultrasound index.	Dinoprostone (50%), Oxytocin (15%), or Dinoprostone + Oxytocin (35%).	Vaginal delivery occurred in the majority. Bishop score (AUC 0.784) vs. ultrasound score (AUC 0.891).	Vaginal (majority), Cesarean (for induction failure or failure to progress).	The new ultrasound score (integrating cervical length, fetal position, elastography, and angulation) was superior to the Bishop Index in predicting vaginal delivery.
Ramsey et al., 2003 [42]	Comparative efficacy and cost of the prostaglandin analogs dinoprostone and misoprostol as labor preinduction agents Level 1.c	Prospective randomized clinical trial	To compare the efficacy and cost of misoprostol, dinoprostone gel, and dinoprostone pessary.	111 pregnant women with unfavorable cervix (Bishop ≤ 5).	Initial Bishop Index, BMI, parity, and CTG monitoring.	Misoprostol (50 µg/6h), Dinoprostone gel (0.5 mg/6h), or Dinoprostone pessary (10 mg).	Induction-to-delivery interval: 24.0 h (Misoprostol) vs. 31.6 h (Gel) vs. 32.2 h (pessary). Mean Bishop change: 5.2 (M) vs. 2.2 (G) vs. 3.2 (I).	Cesarean (12.6% overall; no significant difference between groups).	Misoprostol is more effective at cervical ripening, faster to delivery, and more cost-effective.
Rayburn et al., 1992 [43]	An Intravaginal Controlled-Release Prostaglandin E2 Pessary for Cervical Ripening and Initiation of Labor at Term Level 1.c	Randomized, double-blind, placebo-controlled study	To evaluate the efficacy and safety of the controlled-release PGE2 pessary.	215 pregnant women (101 PGE2, 114 placebo) with Bishop ≤ 4 at term.	Bishop Index ≤ 4, age ≥ 18 years, singleton cephalic pregnancy.	10 mg controlled-release vaginal PGE2 pessary vs. Placebo for 12 h.	Bishop Index increase ≥ 3 in 59% (PGE2) vs. 18% (Placebo). Treatment success (Bishop ≥ 6 or vaginal delivery) in 68% (nulliparous PGE2).	Vaginal delivery (PGE2: 15% at 12 h vs. 0.9% placebo), Cesarean (28% PGE2 vs. 36% placebo).	The controlled-release PGE2 pessary produces appreciable cervical ripening and frequently initiates labor without the need for oxytocin.
Uyar et al., 2009 [44]	Comparison of the Bishop score, body mass index and transvaginal cervical length in predicting the success of labor induction Level 3.e	Prospective observational follow-up study	To assess the role of BMI, cervical length, and Bishop Index in predicting induction success.	189 pregnant women (37–42 weeks) undergoing oxytocin induction.	BMI, cervical length (transvaginal ultrasound), and Bishop Index.	Oxytocin (initial dose 2 mIU/min, doubled every 10 min).	Vaginal delivery in 83.1%. Cesarean in 16.9%. Cervical length was the best predictor (AUC 0.819).	Vaginal (83.1%), Cesarean (16.9%).	Cervical length and BMI were better predictors of induction success than the Bishop Index.
Wise et al., 2023 [45]	Outpatient balloon catheter vs. inpatient prostaglandin for induction of labor: a randomized trial Level 1.c	Multicenter superiority randomized clinical trial	To compare induction with outpatient balloon catheter vs. inpatient prostaglandin E2.	1087 pregnant women (539 outpatient balloon, 548 inpatient PGE2).	Modified Bishop Index 0 to 6, intact membranes, term pregnancy.	Outpatient Foley balloon catheter (50 mL) vs. inpatient prostaglandin (dinoprostone gel/pessary).	Vaginal delivery at 24 h: 13.4% (balloon) vs. 31.2% (PGE2). Time in hospital before delivery: 12 h less in the balloon group.	Cesarean (41.0% balloon vs. 35.2% PGE2).	Outpatient balloon induction did not reduce the cesarean rate, but is safe and reduces pre-delivery hospital stay.
Wollmann et al., 2017 [46]	Time-to-delivery and delivery outcomes comparing three methods of labor induction in 7551 nulliparous women: a population-based cohort study Level 3.e	Population-based cohort study	To determine time-to-delivery and delivery type across three induction methods	7551 nulliparous women with singleton pregnancy ≥ 37 weeks and Bishop ≤ 6.	Bishop Index ≤ 6, parity (all nulliparous), term pregnancy.	Dinoprostone, Misoprostol, or single transcervical balloon catheter.	Mean time to delivery: 18.3 h (Balloon), 23.7 h (Misoprostol), 25.2 h (Dinoprostone). Delivery at 24 h: 95% (Balloon) vs. 55% (M) vs. 54% (D).	Cesarean (no significant differences between methods), Instrumental vaginal delivery (higher with misoprostol).	The balloon catheter is the most effective method for reducing time to delivery in nulliparous women with an unripe cervix.
Wu et al., 2025 [47]	Comparison of an Ultrasound-Based System and the Bishop Score for Predicting Labor Induction Timing in Singleton Pregnancies at Term: A Prospective Cohort Study Level 3.e	Prospective observational cohort study	To evaluate the predictive accuracy of the LaborPro system (ultrasound + magnetic tracking) vs. Bishop Index.	156 term pregnant women undergoing induction.	Clinical Bishop Index and LaborPro measurements (cervical length and presentation level).	Oxytocin (if Bishop ≥ 6), PROPESS or Cook catheter (if Bishop < 6).	Vaginal delivery at 24 h (VD24) in 53.8% of those who delivered vaginally. Median time: 779.5 min (VD24) vs. 1884.5 min (Non-VD24).	Vaginal delivery (analysis focused on the 156 who delivered vaginally).	Objective measurements of cervical length and fetal presentation level significantly outperform the Bishop Index in predicting induction success.

**Table 5:** Overall characteristics of the included studies.

Dimension Analyzed	Summary of the Characterization
Total number of included studies	21
Publication period	1992–2026
Temporal distribution	1992–2009: 7 studies 2010–2019: 6 studies 2020–2026: 8 studies
Geographic origin	11 countries: United States, China, Japan, Germany, France, Israel, Spain, Greece, Italy, the Netherlands, and Turkey
JBI levels of evidence	Level 1.c: 6 studies Level 3.e: 11 studies Level 4.b: 4 studies
JBI methodological quality	High (≥80%): 19 studies Moderate (≥75%): 2 studies
Range of JBI scores	76.9–100%

## Data Availability

No new data were created or analyzed in this study. Data sharing is not applicable to this article.

## Supplementary Materials

The following supporting information can be downloaded at https://www.mdpi.com/article/10.3390/healthcare14152242/s1, Table S1 presents Methodological quality appraisal of the 38 eligible studies. Refs. [16,17,29,30,31,32,33,34,35,36,37,38,39,40,41,42,43,44,45,46,47,49,50,51,52,53,54,55,56,57,58,59,60,61,62,63,64,65] are cited in the Supplementary Materials.

## Abbreviations

The following abbreviations are used in this manuscript:

AUC	Area Under the Curve
CINAHL	Cumulative Index to Nursing and Allied Health Literature
EBSCO	Elton B. Stephens Company
JBI	Joanna Briggs Institute
MEDLINE	Medical Literature Analysis and Retrieval System Online
OR	Odds Ratio
OSF	Open Science Framework
PCC	Population, Concept, Context
PRISMA-ScR	Preferred Reporting Items for Systematic reviews and Meta-Analyses extension for Scoping Reviews
PUBMED	Public MEDLINE
RCT	Randomized Controlled Trial
