# Supplementary material for: The Bishop Index and Birth Outcomes in Nulliparous Women Undergoing Labor Induction: A Scoping Review
Source: Healthcare (Basel). 2026 Jul 23;14(15):2242. doi: 10.3390/healthcare14152242 (PMC13465716; doi:10.3390/healthcare14152242)
Supplement: Supplementary file 1 [file healthcare-14-02242-s001.zip › healthcare-4396010-supplementary.pdf]

healthcare

MDPI

Table S1. Methodological quality appraisal of the 38 eligible studies*.

Author Year	Country	Type of Study	Tool JBI	Absolute score	Applicable criteria	Score (%)	Classification
Agarwal et al., 2003[49]	India	Randomized clinical trial	JBI RCT	7	13	53.8%	Low
Chyu et al., 1997[50]	United States	Randomized clinical trial	JBI RCT	7	13	53.8%	Low
Koc et al., 2013[51]	Turkey	Randomized clinical trial	JBI RCT	7	13	53.8%	Low
Ottinger et al., 1998[52]	United States	Randomized clinical trial	JBI RCT	7	13	53.8%	Low
Qamar et al., 2012[53]	Pakistan	Quasi-experimental	JBI Quasi-experimental	5	9	55.6%	Low
Parazzini et al., 1998[54]	Italy	Multicenter randomized clinical trial	JBI RCT	8	13	61.5%	Moderate
Triglia et al., 2010[55]	Italy	Randomized clinical trial	JBI RCT	8	13	61.5%	Moderate
Williams et al., 1997[56]	USA	Randomized clinical trial	JBI RCT	8	13	61.5%	Moderate
Wing et al., 1997[57]	USA	Randomized clinical trial	JBI RCT	8	13	61.5%	Moderate
Ducarme et al., 2023[58]	France	Prospective comparative cohort	JBI Coorte	7	11	63.6%	Moderate
Ikeotuonye et al., 2018[59]	Nigeria	Retrospective cohort	JBI Coorte	7	11	63.6%	Moderate
Oliveira et al., 2011[60]	Portugal	Retrospective comparative cohort	JBI Coorte	7	11	63.6%	Moderate
Silfeler et al., 2011[61]	Turkey	Quasi-experimental	JBI Quasi-experimental	6	9	66.7%	Moderate
Wang et al., 2020[62]	China	Quasi-experimental	JBI Quasi-experimental	6	9	66.7%	Moderate
Marconi et al., 2008[63]	Italy	Randomized clinical trial	JBI RCT	9	13	69.2%	Moderate
Wang et al., 2016[64]	China	Randomized clinical trial	JBI RCT	9	13	69.2%	Moderate
Wei et al., 2019[65]	China	Multicenter retrospective cohort	JBI Coorte	8	11	72.7%	Moderate
Rayburn et al., 1992[43]	USA	Randomized clinical trial	JBI RCT	10	13	76.9%	Moderate
Wise et al., 2023[45]	USA	Multicenter randomized clinical trial	JBI RCT	10	13	76.9%	Moderate

Gonen et al., 1998[35]	Israel	Prospective cohort	JBI Coorte	8	10	80.0%	High
Tollon et al., 2023[32]	France	Multicenter prospective cohort	JBI Coorte	8	10	80.0%	High
Uyar et al., 2009[44]	Turkey	Prospective cohort	JBI Coorte	8	10	80.0%	High
Zhao et al., 2019[17]	China	Retrospective cohort	JBI Coorte	8	10	80.0%	High
Ashwal et al., 2017[29]	Israel	Retrospective cohort	JBI Coorte	9	11	81.8%	High
Droulez et al., 2008 [33]	France	Prospective cohort	JBI Coorte	9	11	81.8%	High
Imai et al., 2023[36]	Japan	Comparative retrospective cohort	JBI Coorte	9	11	81.8%	High
Imai et al., 2023[37]	Japan	Retrospective cohort	JBI Coorte	9	11	81.8%	High
Mayer et al., 2016[39]	Germany	Comparative retrospective cohort	JBI Coorte	9	11	81.8%	High
Wollmann et al., 2017[46]	Germany	Population cohort	JBI Coorte	9	11	81.8%	High
Ramsey et al., 2003[42]	USA	Randomized clinical trial	JBI RCT	11	13	84.6%	High
Chen et al., 2014[30]	China	Quasi-experimental	JBI Quasi-experimental	8	9	88.9%	High
López-Jiménez et al., 2023[16]	Spain	Predictive prospective cohort	JBI Coorte	9	10	90.0%	High
Meijer-Hoogeveen et al., 2009[40]	Netherlands	Predictive prospective cohort	JBI Coorte	9	10	90.0%	High
Gerli et al., 2013[34]	Italy	Retrospective cohort	JBI Coorte	10	10	100.0%	High
Itoh et al., 2021[38]	Japan	Randomized clinical trial	JBI RCT	13	13	100.0%	High
Michail et al., 2026[41]	Greece	Predictive prospective cohort	JBI Coorte	10	10	100.0%	High
Sherman et al., 2001[31]	USA	Double-blind randomized clinical trial	JBI RCT	13	13	100.0%	High
Wu et al., 2026[47]	China	Predictive prospective cohort	JBI Coorte	10	10	100.0%	High

* Table created in accordance with the JBI’s critical appraisal tools, for each type of study.

https://doi.org/10.3390/xxxxx
